# Comparison of percutaneous closure systems for large bore vascular access sites in endovascular procedures

**DOI:** 10.3389/fcvm.2023.1130627

**Published:** 2023-04-05

**Authors:** Luka Košak, Davorka Lulić, Tomislav Jakljević, David Gobić, Josip Aničić, Vjekoslav Tomulić

**Affiliations:** ^1^ Institute of Emergency Medicine of Istra County, Pula, Croatia; ^2^ Clinic for Cardiovascular Diseases, Clinical Hospital Center Rijeka, Rijeka, Croatia

**Keywords:** bleeding, bleeding academic research consortium (BARC), endovascular aortic repair, valve academic research consortium-3 (VARC-3), thoracic endovascular aortic repair, transcatheter aortic valve replacement, vascular closure device

## Abstract

**Backgrounds:**

The vascular closure device (VCD) is a medical device used for achieving hemostasis of vascular access sites greater than 8 Fr. We compared complications after placement of Perclose ProGlide (Abbott Vascular, USA), a percutaneous suture-mediated closure system, with MANTA VCD (Teleflex Vascular, USA), a collagen-based closure device.

**Methods:**

This retrospective cohort study analyzed procedures performed between 2016 and 2021. We compared the incidence of bleeding complications according to the Bleeding Academic Research Consortium (BARC) and Valve Academic Research Consortium-3 (VARC-3) criteria. The comparison was made between two cohorts of patients: in the first, vascular access sites were closed with a double Perclose ProGlide system, and in the second with an 18 Fr MANTA VCD.

**Results:**

A total of 189 patients were included in the study, out of which 63% were male and 37% were female, with a median age of 79 (72–83) years. All devices were used for femoral arterial access closure. A double Perclose ProGlide was used in 91 (48%) patients, while MANTA VCD was used in 98 patients (52%). The distribution of patients by VARC-3 and BARC bleeding criteria differs between groups (*p* = 0.017). A significantly higher incidence of VARC 1 (14% vs. 4%; *p* = 0.020) and BARC 1–2 (14% vs. 4%; *p* = 0.020) complications in the Perclose ProGlide cohort was observed. VARC 3 (1% vs. 5%; *p* = 0.213) and BARC 3b (1% vs. 5%; *p* = 0.213) complications showed higher, but statistically non-significant rates of major bleeding complications in the MANTA VCD cohort. The need for subsequent surgical revision did not show a significant difference between the cohorts (2% vs. 6%; *p* = 0.281).

**Conclusion:**

The Perclose ProGlide cohort was associated with a significantly higher rate of milder complications. MANTA VCD cohort had a higher rate of major bleeding complications, requiring more complex treatment with a potentially larger impact on quality of life.

## 1. Introduction

The vascular closure device (VCD) is a device that can achieve large bore access (>8 Fr) hemostasis after transcatheter aortic valve replacement (TAVR) and endovascular repair of abdominal and thoracic aortic aneurysms (EVAR, TEVAR) (1, 2).

Perclose ProGlide^TM^ Suture-Mediated Closure (SMC) System is a suture-mediated percutaneous endovascular closure system, classified as an active approximator. The vascular access larger than 8 Fr should be closed with two systems oriented towards 10 h and 2 h and can be used for hemostasis of the arterial access sites up to 21 Fr (3). VCDs should be deployed before the introduction of a large bore access sheath. The system is based on the application of needles, followed by the attachment and withdrawal of the sutures through the same path in the arterial wall formed by the needles. If the suture is safely deployed, the arteriotomy can be closed and the node secured (4).

MANTA VCD is a passive approximator used for femoral access site closure following the deployment of sheaths up to 25 Fr outer diameter. The hemostasis is based on collagen-stimulated coagulation. The system is deployed at a 45° angle. The anchor is released by rotating the lever, followed by the withdrawal of the device that causes the anchor-wall interaction, the release of the collagen plaque, and safe arteriotomy closure (5).

VCDs have significantly reduced the incidence of complications compared to surgical closure (6), but bleeding complications in Perclose ProGlide and MANTA VCD patients are still often and can occur in up to 33% and 18% of cases (7–10). The severity of bleeding complications is defined by the Bleeding Academic Research Consortium (BARC) and the Valve Academic Research Consortium-3 (VARC-3) criteria, which enable us to adequately compare efficiency and safety (Table 1) (11, 12). Several studies have been published with opposing results on the safety of discussed VCDs. We gathered the results focusing on complications defined by both the BARC and VARC-3 bleeding criteria.

**Table 1:** VARC-3 criteria for bleeding and transfusion.

VARC 1	• Overt bleeding that does not require surgical or percutaneous intervention, but does require medical intervention by a health care professional, leading to hospitalization, an increased level of care, or medical evaluation **(BARC 2)** • Overt bleeding that requires a transfusion of 1 unit of whole blood/red blood cells **(BARC 3a)**
VARC 2	• Overt bleeding that requires a transfusion of 2–4 units of whole blood/ red blood cells **(BARC 3a)** • Overt bleeding associated with a haemoglobin drop of >3 g/dl (>1.86 mmol/L) but <5 g/d (<3.1 mmol/L) **(BARC 3a)**
VARC 3	• Overt bleeding in a critical organ, such as intracranial, intraspinal, intraocular, pericardial (associated with haemodynamic compromise/tamponade and necessitating intervention), or intramuscular with compartment syndrome **(BARC 3b, BARC 3c)** • Overt bleeding causing hypovolemic shock or severe hypotension (systolic blood pressure <90 mmHg lasting >30 min and not responding to volume resuscitation) or requiring vasopressors or surgery **(BARC 3b)** • Overt bleeding requiring reoperation, surgical exploration, or reintervention for the purpose of controlling bleeding **(BARC 3b, BARC 4)** • Post-thoracotomy chest tube output $2 L within a 24-h period **(BARC 4)** • Overt bleeding requiring a transfusion of $5 units of whole blood/red blood cells **(BARC 3a)** • Overt bleeding associated with a haemoglobin drop $5 g/dl ($3.1 mmol/L) **(BARC 3b)**
VARC 4	• Overt bleeding leading to death. Should be classified as: • Probable: Clinical suspicion **(BARC 5a)** • Definite: Confirmed by autopsy or imaging **(BARC 5b)**

Adapted from: Généreux P, Piazza N, Alu MC, Nazif T, Hahn RT, Pibarot P, et al. Valve academic research consortium 3: updated endpoint definitions for aortic valve clinical research. J. Am. Coll. Cardiol. 2021 Jun;**77**(21):2717–46.

## 2. Materials and methods

Our retrospective cohort study was conducted at the Clinic for Cardiovascular Diseases, Clinical Hospital Center Rijeka, Croatia. Patients undergoing TAVR, EVAR, and TEVAR procedures followed by hemostasis of the femoral access site achieved with the Perclose ProGlide or MANTA VCD, between 2016 and 2021 were analyzed. Cohorts were determined based on the vascular closure device application. The femoral access site in the first cohort was closed with a double Perclose ProGlide system, while a single 18 Fr MANTA VCD was used in the second cohort.

### 2.1. Statistical analysis

Data were collected in Microsoft Access 365 database (Microsoft Inc., Redmond, Washington, USA), while the statistical analysis was performed in Statistica 14.0 (TIBCO Software Inc., Palo Alto, California, USA). Categorical variables were shown as a percentage (%) and statistically processed using the chi-square test or Fisher exact test. The Kolmogorov-Smirnov test determined the distribution of continuous data. Variables with normal distribution were presented as arithmetic means (± standard deviation), and the student *T*-test was used to determine the difference between cohorts. The median (interquartile range/Q3–Q1/) was used in abnormally distributed data followed by Mann Whitney *U*-test. A bidirectional *p*-value < 0.05 was considered as a significant difference between the compared variables.

### 2.2. Procedures

All patients were presented to a multidisciplinary team and underwent CT angiography to rule out extreme tortuosity and calcifications at the vascular access site as a routine preoperative workup. At least 5,000 IU of heparin was administered to all patients. Activated coagulation time (ACT) was measured 15 to 20 min after heparin administration, aiming for a value of over 200 s.

Experienced endovascular operators performed the procedures with vascular surgeons on standby in case of complications. The arterial puncture was fluoroscopy guided. The procedure ended with a verification of the efficiency of hemostasis assessed by digital subtraction angiography (DSA). All of the patients had systolic blood pressure lower than 120 mmHg (managed with intravenous nitroglycerin) at the time of the closure device application, irrespective of the type of anesthesia used. ACT was measured just before closure time, and values were <200 s in all patients. Heparin antidote protamine was used only once (0.5%) according to the indication and judgment of the operator.

### 2.3. Variable outcomes

The main observed variable was bleeding, classified according to BARC and VARC-3 bleeding criteria. The BARC 1 and BARC 2 categories were unified as BARC 1–2, while the BARC 4 category was excluded from the study due to its association with the coronary artery bypass graft (CABG) procedure. Bleeding complications of vascular access sites during the procedure were treated with either surgical revision, balloon angioplasty, stent-graft implantation, or with the additional placement of an Angioseal VCD (Terumo Corp., Japan). If the bleeding during the procedure was successfully treated and the postoperative period was without complications, the endovascular procedure was classified as BARC 0 but was not statistically analyzed, while according to the VARC-3 criteria, it was not taken into consideration. Also, subsequent surgical revision was indicated during hospitalization in patients with symptoms and signs of bleeding even after the successful closure of the percutaneous vascular access site. Overall hospitalization duration and Intensive Care Unit (ICU) stay length were taken into consideration. If the patient underwent other procedures or was treated for a different condition during the same stay, the length of hospitalization was not excluded from the study.

## 3. Results

The study included a total of 189 patients in whom hemostasis of vascular access sites was achieved with a double Perclose ProGlide (*n* = 91) or with a single 18 Fr MANTA VCD (*n* = 98). The median age of the whole study sample was 79 (72–83) with 63% of males and 37% of females. Patients in whom MANTA VCD was used were significantly older compared to the Perclose ProGlide cohort (77/69–82/ vs. 80/75.3–84/; *p* = 0.005) (Table 2). However, the MANTA VCD cohort had significantly higher platelet values at admission (187.1 ± 61.5 vs. 211 ± 67.7; *p* = 0.019) and significantly higher values of the lowest platelet count during hospitalization (Pt min; 133.3 ± 52.2 vs. 149 ± 53.6; *p* = 0.045) (Table 2).

**Table 2:** Clinical and procedural characteristics.

	All (*n* = 189)	Perclose ProGlide (*n* = 91)	MANTA (*n* = 98)	*p*-values
**General data and comorbidities**				
Male/Female	119 (63)/70 (37)	60 (66)	59 (60)	0.415
Age (years)	79 (72–83)	77 (69–82)	80 (75,3–84)	**0** **.** **005**
BMI (kg/m^2^)	27,2 ± 4,3	27,3 ± 4,5	27,1 ± 4,1	0.617
BMI > 30 (kg/m^2^)	40 (21)	22 (24)	18 (18)	0.424
DM	53 (28)	26 (29)	27 (28)	0.876
AH	157 (83)	74 (81)	83 (85)	0.536
HLP	86 (46)	35 (38)	51 (52)	0.061
CKD	37 (20)	15 (16)	22 (22)	0.302
AF	62 (33)	24 (26)	38 (39)	0.070
CVD	32 (17)	15 (16)	17 (17)	0.874
PAD	11 (6)	7 (8)	4 (4)	0.289
KOBP	14 (7)	10 (11)	4 (4)	0.700
pMI	28 (15)	16 (18)	12 (12)	0.302
pPCI	37 (20)	15 (16)	22 (22)	0.302
pCABG	14 (7)	6 (7)	8 (8)	0.681
**Laboratory findings and length of hospitalization**				
Hgb admission (g/L)	125,2 ± 17,5	124,3 ± 18,1	125,9 ± 16,9	0.557
Hgb after procedure (g/L)	113,8 ± 16	112,9 ± 15,9	114,6 ± 16,2	0.465
Drop of Hb after procedure (g/L)	11,9 ± 11,9	12,4 ± 12,6	11,5 ± 11,4	0.624
Hgb discharge (g/L)	111,8 ± 14,3	109,8 ± 13,9	113,6 ± 14,4	0.077
Pt admission (10^9^/L)	199,3 ± 65,7	187,1 ± 61,5	211 ± 67,7	**0**.**019**
Pt min (10^9^/L)	141,6 ± 53,4	133,3 ± 52,2	149 ± 53,6	**0**.**045**
Pt at discharge (10^9^/L)	150,5 (123,5–217,3)	146 (119,5–235)	152,5 (129,3–207)	0.653
Crea admission (µmol/L)	94 (74–117)	93 (74,75–113)	96 (74–118)	0.704
Crea max (µmol/L)	104 (83–134)	100.5 (85–127,3)	105 (78–140)	0.646

Values are: frequency (percentage/%/), arithmetic mean (±standard deviation) or median (interquartile range/Q1–Q3/).

BMI, body mass index; DM, diabetes mellitus; AH, arterial hypertension; CKD, chronic kidney disease; AF, atrial fibrillation; CVD, cerebrovascular disease; PAD, peripheral arterial disease; COPD, chronic obstructive pulmonary disease; pMI, previous myocardial Infarction; pPCI, previous percutaneous coronary intervention; pCABG, previous coronary artery bypass graf; Hgb, hemoglobin; Pt, platelets; Crea, creatinine.

There was no statistically significant difference between the cohorts in the ICU and overall hospital length of stay (Table 3). Statistical analysis of anesthesia procedures showed a significantly higher rate of local anesthesia in the Perclose ProGlide cohort (34% vs. 10%; *p* < 0.001), while in the MANTA VCD cohort, local anesthesia and sedation (55% vs. 86%, *p* < 0.001) was used significantly more often (Table 4).

**Table 3:** Length of hospitalization.

	All (*n* = 189)	Perclose ProGlide (*n* = 91)	MANTA VCD (*n* = 98)	*p*-values
Hospital stay (days)	7 (64–1) (5–12)	7 (5,5–12)	6 (5–11,8)	0.067
ICU stay (days)	2 (58–1) (2–5)	3 (2–5)	2 (2–)	0.110

Values are: median (interquartile range/Q1–Q3/).

ICU, intensive care unite.

**Table 4:** Anticoagulant and antiplatelet therapy and types of anesthesia.

	All (%) (*n* = 189)	Perclose ProGlide (%) (*n* = 91)	MANTA (%) (*n* = 98)	*p*-values
Antiplatelet therapy (admission)				1
No	115 (60)	55 (60)	60 (61)	0.912
Yes	74 (39)	36 (40)	38 (39)	0.912
ASA	66 (35)	32 (35)	34 (35)	0.945
Dual	8 (4)	4 (4)	4 (4)	1
Anticoagulant therapy (admission)				0.118
No	138 (73)	72 (79)	66 (67)	0.068
Yes	51 (27)	19 (21)	32 (33)	0.068
Warfarin	22 (12)	10 (11)	12 (12)	0.788
NOAC	29 (15)	9 (10)	20 (20)	**0** **.** **045**
Antiplatelet therapy (discharge)				0.082
ASA	34 (18)	22 (24)	12 (12)	**0**.**033**
ASA + clopidogrel	75 (40)	35 (38)	40 (41)	0.741
NOAC/warfarin	59 (31)	23 (25)	36 (37)	0.089
NOAC/warfarin and clopidogrel	12 (6)	4 (4)	8 (8)	0.376
**Types of anesthesia**				
Local anesthesia	41 (22)	31 (34)	10 (10)	**<0**.**001**
Local anesthesia and sedation	134 (71)	50 (55)	84 (86)	**<0**.**001**
General anesthesia	14(7)	10(11)	4(4)	0.095

Value is: frequency (percentage/%/).

No, without therapy; ASA, acetylsalicylic acid; Dual, ASA + P2Y12 inhibitor; NOAK, novel oral anticoagulants.

The patient's distribution differs between cohorts by BARC (*p* = 0.017) and VARC-3 (*p* = 0.017) criteria for bleeding. Statistical analysis of each independent BARC and VARC-3 bleeding criteria showed significantly more BARC 1–2 and VARC 1 complications in the Perclose ProGlide cohort (14% vs. 4%; *p* = 0.020). Aside from the exclusion of BARC 4 criteria, the results highlighted that there were no deaths associated with VCDs (BARC 5, VARC 4), while the proportions of patients in other BARC and VARC-3 categories did not show significant differences (Table 5).

**Table 5:** Complications.

	All (%) (*n* = 189)	Perclose ProGlide (%) (*n* = 91)	MANTA (%) (*n* = 98)	*p*-values
Complications				**<0** **.** **001**
Surgical revision	2 (1)	0 (0)	2 (2)	0.498
Balloon dilatation	4 (2)	0 (0)	4 (4)	0.122
Stent graft implantation	2 (1)	0 (0)	2 (2)	0.498
Angioseal VCD	9 (5)	9 (10)	0 (0)	**0**.**001**
Subsequent surgical revision	8 (4)	2 (2)	6 (6)	0.281
**BARC**				**0**.**017**
BARC 1–2	17 (9)	13 (14)	4 (4)	**0**.**020**
BARC 3a	3 (2)	1 (1)	2 (2)	1
BARC 3b	6 (3)	1 (1)	5 (5)	0.213
BARC 5	0 (0)	0 (0)	0 (0)	1
**VARC**				**0**.**017**
VARC 1	17 (9)	13 (14)	4 (4)	**0**.**020**
VARC 2	3 (2)	1 (1)	2 (2)	1
VARC 3	6 (3)	1 (1)	5 (5)	0.213
VARC 4	0(0)	0(0)	0(0)	1

Value is: frequency (percentage/%/).

Complications, correction of complications during the procedure; BARC, bleeding academic research consortium; VARC, valve academic research consortium.

Four patients in the MANTA VCD cohort were successfully treated with balloon catheter dilatation: three because of bleeding and one patient had acute occlusion at the puncture site (Table 5).

VARC 1 bleeding complications were significantly more frequent after EVAR/TEVAR procedures in patients in whom Perclose ProGlide was used (6% vs. 14%; *p* = 0.036), while other results did not show the significant difference in proportions between examined groups. It is important to highlight the considerably smaller EVAR/TEVAR sample in the MANTA VCD cohort (Table 6).

**Table 6:** VARC-3 bleeding complications comparison by the type of procedures.

	TAVR (*n* = 128)	EVAR/TEVAR (*n* = 61)	*p*-value
**Perclose ProGlide**	***n* = 47 (37)**	***n* = 44 (72)**	
VARC 1	3 (6)	10 (14)	**0**.**036**
VARC 2	1 (2)	0 (0)	1
VARC 3	0 (0)	1 (1)	0.484
VARC 4	0 (0)	0 (0)	1
**MANTA VCD**	***n* = 81** (63)	***n* = 17** (28)	
VARC 1	3 (4)	1 (6)	0.539
VARC 2	2 (2)	0 (0)	0.540
VARC 3	5 (6)	0 (0)	0.161
VARC 4	0(0)	0(0)	1

Value is: frequency (percentage/%/).

TARV, transcatheter aortic valve replacement; EVAR, endovascular aneurysm repair; TEVAR, thoracic endovascular aortic repair.

When comparing by gender, there were no significant differences in the length of hospitalization stay and severity of complications (male vs. female, Table 7).

**Table 7:** Comparison of hospital stay and VARC-3 bleeding complications by gender.

	Female (*n* = 70)	Male (*n* = 119)	*p*-value
Hospital stay (days)	6 (5–11)	7 (5–12)	0.177
**VARC**			
VARC 1	8 (11)	9 (3)	0.370
VARC 2	0 (0)	3 (3)	0.297
VARC 3	2 (3)	4 (3)	1
VARC 4	0 (0)	0(0)	1

Value is: frequency (percentage/%/), median (interquartile range/Q1–Q3/).

VARC, valve academic research consortium.

## 4. Discussion

**Figure 1:**
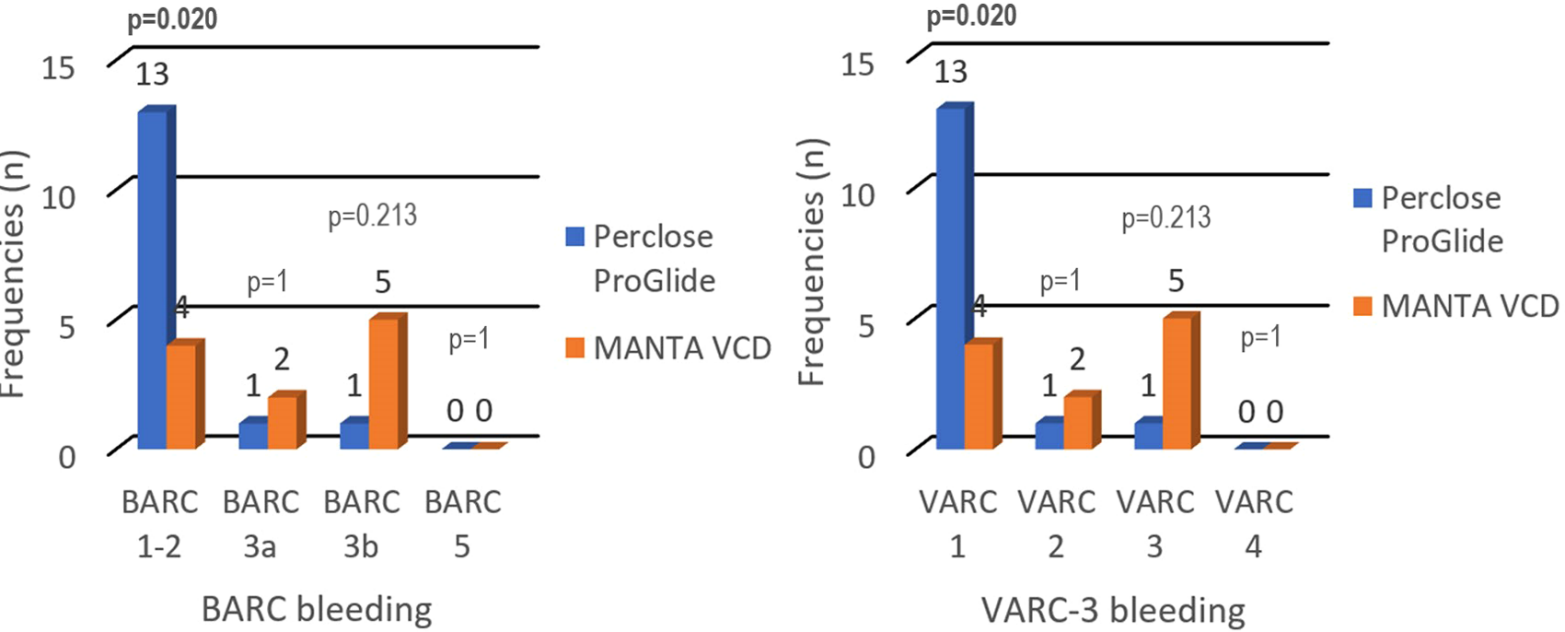
BARC and VARC-3 bleeding criteria—Perclose ProGlide vs. MANTA VCD.

Bleeding during and after endovascular procedures significantly impacts the outcome and quality of a patient's life. To our knowledge, this is the first retrospective study comparing the performance of Perclose ProGlide and MANTA VCDs according to both BARC and VARC-3 bleeding criteria. Our study concluded that there is a significant difference in patient distribution between cohorts according to VARC-3 (*p* = 0.017) and BARC (*p* = 0.017) bleeding criteria, but neither showed unidirectional results while analyzing each independent category. The difference in BARC and VARC-3 bleeding criteria is not seen due to the BARC 1 and BARC 2 categories being unified as BARC 1–2 and as a result, the frequencies became equal. Assessing the frequency of certain types of complications, the Perclose ProGlide system has a significantly higher rate of milder complications (VARC 1; BARC 1–2), while the MANTA VCD has a numerically higher, but statistically insignificant rate of more severe complications (VARC 2; VARC 3; BARC3a; BARC 3b) that require more challenging treatment with potentially poorer quality of life and higher mortality in the upcoming years (Figure 1). It should be noted that the number of patients in some categories is small, which should be taken into consideration while interpreting the results. Also, there were no deaths associated with the closure of vascular access sites, but seven patients (3.7%) died from other causes (cardiogenic shock, tamponade, and stroke). Patients who died during the hospitalization, but with successful closure of vascular access sites, were not classified in the BARC and VARC-3 criteria.

The differences in the definitions of VARC-2 and the revised VARC-3 bleeding criteria should be taken into consideration while assessing and comparing the results with other studies. The main difference between the criteria is in the number of used units of whole blood/red blood cells. BARC classifies any blood transfusion as BARC 3a, while VARC classification divides it by the number of units used. In our study, all the patients who received between two and four blood transfusion units had also an indication for surgical revision and were therefore classified as VARC 3 instead of VARC 2.

A high percentage of the heterogeneity of results is present in the studies published so far. There have been studies in which the MANTA VCD is non-significantly more effective than the Perclose ProGlide closure system in comparing minor (7, 8), major or life-threatening complications (13), and significantly more effective in comparing major (7, 8) and life-threatening complications (7) of the VARC-2 bleeding criteria. On the other hand, Abdel-Wahab et al. (14) published results in which Perclose ProGlide showed better but non-significant results in every VARC-2 bleeding category, while van Wiechen et al. (15) found no significant difference while comparing Perclose ProGlide and MANTA VCD based on the variable defined as any access site bleeding within 30 days.

The Perclose ProGlide cohort's significantly lower platelet counts per admission and lower minimum platelet count could be the cause of the higher frequency of VARC 1 complications, which may complicate primary coagulation (platelet plug formation) at the puncture site and may interact with complete hemostasis. Also, the MANTA VCD anchor fits better on the arterial wall, does not depend on the suture tension, and is easier to use, so it could be less dependent on the operator's experience. Significantly older patients in the MANTA VCD cohort often imply a greater number of more serious comorbidities, which may be the cause of an insignificantly higher incidence of VARC 3 bleeding (Table 2). No matter which VCD was used, there was no clear impact on the duration of ICU stay and overall hospitalization length, also when comparing genders (Tables 3, 7).

Several studies published the results of the VCD success ratio according to the BARC or VARC criteria for bleeding but did not compare both Perclose ProGlide and MANTA VCD. Most published studies define VARC bleeding criteria according to the older, VARC-2 definition, so comparing results defined by different criteria with complete certainty is not possible. The Perclose ProGlide studies coincide with our study and all show a higher rate of milder bleeding complications regardless of the criteria definition (16, 17). Eckner et al. (17) published the results according to BARC criteria as major (type 3a and 3b) in 2.4% and minor (type 1 and 2) in 16.9%. They also reported one case of death (BARC 5) that did not occur in our research cohorts. The MANTA VCD studies showed that the incidence of minor complications varied up to 14%, the incidence of major complications was up to 3%, and life-threatening complications was up to 4% according to the VARC-2 criteria for bleeding, which is comparable to our results (9, 10, 18–20). It is necessary to point out the study by Maisero et al. (21), published in February 2022 as the only research in which the frequency of bleeding complications was classified according to the newest VARC-3 criteria, used in this study. The results showed that the frequencies of mild VARC 1 bleeding complications were higher (14% vs. 4%), while the results in other categories roughly correspond with our study (VARC 2: 1% vs. 2%; VARC 3–4: 2% vs. 5%).

Table 5 describes treatments used to achieve hemostasis during the procedure in case of bleeding complications or failure of the closure devices. All intraoperative bleeding complications after the application of Perclose ProGlide were successfully corrected with an Angioseal VCD. The Angioseal VCD could not be used in the MANTA VCD cohort due to the limitation of applying two collagen-based systems in the same arteriotomy. Unsuccessful applications of the MANTA VCDs were repaired with temporary balloon dilation in four cases (4%; three cases of bleeding, and one case of acute vessel occlusion), while more significant bleeding complications were permanently stopped in two cases (2%) by placing a stent graft at the bleeding site previously determined by angiography. Balloon angioplasty hemostasis was attempted in one MANTA VCD patient, with suboptimal results, followed by successful stent-graft placement. Surgical revision during the procedure was indicated if other endovascular hemostatic methods could not achieve hemostasis. It was performed in two patients (2%) closed with the MANTA VCD. On the other hand, the need for a later, subsequent surgery is potentially the most reliable success indicator for vascular closure devices. Any need for subsequent surgical revision is considered an unsuccessful VCD placement with uncontrolled hemorrhage and potential development of hypotensive shock. Our study showed that the Perclose ProGlide cohort had an insignificantly lower rate of subsequent surgical revision (2% vs. 6%; *p* = 0.281), which is expected considering the MANTA VCD cohort showed more VARC 3 complications. Other studies have shown various results, Moriyama et al. (8) reported the need for subsequent surgical revision in 7% of each, Perclose ProGlide and MANTA VCD cohort. Moreover, Moccetti et al. (9) also reported the need for subsequent surgical revision in 7% of cases, but only for the MANTA VCD cohort, while Hoffmann et al. (22) reported that the Perclose ProGlide and MANTA VCD cohorts had a 0% necessity for postoperative open surgery and 1.3% necessity for perioperative open surgery. However, they did not specify a time difference between the two terms.

The significantly higher rate of VARC 1 complications in EVAR/TEVAR procedures closed with Perclose ProGlide could be explained by the difference in the size of catheters used in different procedures. TEVAR procedures have wider sheaths of up to 24 Fr, while TAVR and EVAR have up to 18 Fr. 72% of Perclose ProGlides were used in EVAR/TEVAR procedures. TEVAR procedure has the biggest arteriotomy which could follow more difficult VCD closure and a higher incidence of all bleeding complications. It should be noted that although Perclose ProGlide was used in all TEVAR cases, only one patient had serious (VARC 3) bleeding complication (Table 6). EVAR and TAVR patients who were treated with Perclose ProGlide had no serious and showed only milder (VARC 1) bleeding complications (Table 6).

## 5. Study limitations

This is a retrospective single-center study with a relatively small sample. Data were collected in the period from 2016 to 2021. The puncture at the access site was guided with angiography, rather than ultrasound. Also, the period of the patient's postprocedural monitoring was not previously unified, so the length of hospitalization was used as the monitoring period. Lastly, the difference in the size of catheters varied depending on the manufacturer and type of procedure.

## 6. Conclusion

The Perclose ProGlide cohort had a significantly higher rate of milder complications, which did not require any therapy and did not affect the patient's prognosis. MANTA VCD had a numerically higher, but statistically non-significant rate of major complications that required more challenging treatment with potentially poorer prognosis. In conclusion, our study of large bore access closure showed a positive trend toward the safer performance of Perclose ProGlide considering major complications in comparison to MANTA VCD. However, further prospective research based on BARC and VARC-3 criteria, preferably multicentric and with a larger number of participants is needed to support this finding.

## Data Availability

The raw data supporting the conclusions of this article will be made available by the authors, without undue reservation.
